# A visual framework for classifying adaptive design clinical trials using the GATE frame and PICO terminology

**DOI:** 10.1016/j.dialog.2026.100315

**Published:** 2026-05-28

**Authors:** David Lora, Antonio Lalueza Blanco, Guillermo Maestro de la Calle, Ana García Reyne, María Ruíz-Ruigómez, Enrique J. Calderón, Miguel Menéndez-Orenga

**Affiliations:** a Instituto de Investigación Sanitaria del Hospital Universitario 12 de Octubre (imas12), Madrid, Spain; b Spanish Clinical Research Network (SCReN), Madrid, Spain; c Facultad de Estudios Estadísticos, Universidad Complutense de Madrid (UCM), Madrid, Spain; d Servicio de Medicina Interna, Hospital Universitario 12 de Octubre, Madrid, Spain; e Facultad de Medicina, Universidad Complutense de Madrid (UCM), Madrid, Spain; f CIBER de Enfermedades Infecciosas (CIBERINFEC), Madrid, Spain; g Servicio de Medicina Interna, Antimicrobial Stewardship Program, Hospital Universitario 12 de Octubre, Madrid, Spain; h Hospital Universitario 12 de Octubre, Madrid, Spain; i Instituto de Biomedicina de Sevilla, Hospital Universitario Virgen del Rocío, Consejo Superior de Investigaciones Científicas, Universidad de Sevilla, Sevilla, Spain; j Centro de Investigación Biomédica en Red de Epidemiología y Salud Pública (CIBERESP), Madrid, Spain; k Departamento de Medicina, Facultad de Medicina, Universidad de Sevilla, Sevilla, Spain; l Servicio Madrileño de Salud, Centro de Salud La Ventilla, Madrid, Spain

**Keywords:** Adaptive design, Clinical trial, PICO terminology, CONSORT adaptive, COVID-19, Teaching tool, Public health preparedness

## Abstract

An adaptive design of a clinical trial is a trial design that offers pre-planned opportunities to use accumulating trial data to modify aspects of an ongoing trial while preserving the validity and integrity of that trial. The Consolidated Standards Of Reporting Trials (CONSORT) 2025 statements show the minimum information that should be included in the reporting of trials to ensure that trial protocols and trials reports are clear and transparent. Pre-planned interim analysis of adaptive clinical trials can modify the study population, the randomization, the intervention arms, the primary outcome or the clinical trial phase. Such key aspects can be reported using the Adaptive designs CONSORT Extension (ACE) statement, and additionally, conceptualised and represented using the Graphic Appraisal Tool for Epidemiological (GATE) and the PICO terminology (Population, Intervention, Comparison and Outcome). The main purpose of this article is to show how the GATE frame can complement the ACE guidelines by serving as a teaching tool to explain adaptive design clinical trials to a clinical or nonspecialist audience using examples of clinical trials for the prevention and treatment of COVID-19.

## Highlights

• The GATE frame and the PICO terminology can complement the ACE guidelines in explaining adaptive design clinical trials.
• Understanding adaptive designs helps researchers identify the optimal framework to answer their scientific question.
• Scientific transparency enables public and patient access to and participation in research, thereby enhancing population health outcomes.

## 1 Introduction

Clinical trials are studies intended to discover or verify the effects of one or more investigational medicines. Randomised clinical trials, when appropriately designed, conducted, analysed, and reported, are generally considered the highest quality evidence in evaluating healthcare interventions [1]. The need of accelerating the evaluation of new therapies, ensuring that trial participants rights, safety and well-being are protected and that the clinical trial results are credible, has fostered the development of the Adaptive Designs for Clinical Trials (ADCT) [2], [3]. The ADCT is a clinical trial design that offers pre-planned opportunities to use accumulating trial data to modify aspects of an ongoing trial while preserving the validity and integrity of that trial [4], [5], [6], [7], [8]. Scientific guidelines have been published in this respect by the European Medicines Agency (EMA) and the Food and Drug Administration (FDA) [8], [9].

Funders, regulators, research ethics committees/institutional review boards, journal editors, researchers, patients and the public need complete and transparent information on its methods and findings to understand and accurately interpret a trial. Scientific transparency enables public and patient access to, and participation in [10], the research process, thereby contributing to improvements in overall population health. The SPIRIT (Standard Protocol Items: Recommendations for Interventional Trials) statement and CONSORT (Consolidated Standards of Reporting Trials) statement improve the completeness of trial protocols and the quality of reporting of a randomised trial, respectively [1], [11]. Adaptive designs CONSORT Extension (ACE) statement include recommendations for ADCT [5], which is currently pending update to reflect the new CONSORT 2025 guidance [1].

The aspects modified during the process of the clinical trial, as well as the features of the design itself, make it possible to group the different existing ADCT types. This way we can find, in general terms, designs like the seamless design, the continuous reassessment method, the group-sequential, the sample size re-estimation, the population enrichment, the biomarker-adaptive or the adaptive randomization, among others [5], [6], [7], [8], [12], [13]. In other words, ADCTs could modify the phase; the study population, including sample size or patient characteristics; the randomization, including allocation criteria; the intervention arms or the primary outcome during the course of the clinical trial [3], [8].

Different authors have represented graphically the modifications caused by adaptive design clinical trials in particular studies. Thus, we find the graphic representation of the sample size re-estimation in the PHOENIX trial [4], the main target modification in the EXAMINE trial [4], the use of the Response Adaptive Randomization Method in the TROXACITABINE trial [7], or the designs of the platform adaptive trial [14]. Showing specific examples helps understand the concepts in practical cases. However, to our knowledge, there is no a common tool to represent these modifications in a standardized way. The Evidence-Based Medicine Working Group [15] developed the Graphic Appraisal Tool for Epidemiological (GATE), which allows us to graphically conceptualise the design and key elements of the research question into four elements: a triangle at the top indicating the type of participants, a circle to define the intervention (and comparison), a square describing the clinical targeted outcome, and last, the time [15], [16]. The acronym PICO (Population, Intervention, Comparison and Outcome) works as a reminder [16]. Using the GATE frame as a teaching tool in the ADCTs could help interpret and understand the modifications of and benefits from the adaptive design to a clinical or nonspecialist audience [10], [17].

Here we show how the GATE frame can complement the ACE guidelines by serving as a teaching tool to explain adaptive design clinical trials to a clinical or nonspecialist audience using examples of clinical trials for the prevention and treatment of COVID-19.

## 2 Literature search

Definitions of common ADCTs were derived from the literature [2], [3], [5], [6], [8], [12] and are summarized in Table 1.

**Table 1:** Definition of the main ADCTs.

Type of adaptive design	Definition
Group Sequential Method	The group sequential design aims to stop the trial early for safety, futility (i.e. lack of benefit), or efficacy as soon as sufficient evidence is reached to make a reliable conclusion based on the accumulating outcome data of a clinical trial
Sample Size *Re*-Estimation Design	The sample size re-estimation design aims to ensure that the sample size for a trial is appropriate, by estimating design parameters at an interim analysis and using these to recalculate the sample size
Enrichment Adaptive Design	This involves trials with the possibility to select, at an interim analysis, the specific subpopulations that benefit the most from the treatment, optimising the resources on the most promising groups of patients. In such trials, patients are divided in sub-groups according to some biomarker or covariate values, and the treatment's efficacy and safety are assessed in each group and overall at an interim analysis. If, according to predefined criteria, patients from one or several sub-groups appear to benefit more from the experimental drug than others, the recruitment is then restricted to these patients for the rest of the trial.
Response Adaptive Randomization Method	This method modifies the treatment allocation ratio to favour treatments indicating beneficial effects.
Covariate Adaptive Randomization Method	This method is used to balance treatment allocation among covariates, baseline characteristics without compromising randomness.
Seamless Design 2-in-1 Design	It consists in investigating multiple research objectives that are traditionally examined in distinct trial phases, in one trial under a single protocol. The 2-in-1 Design allows an ongoing small-scale trial (e.g. a Phase IIb trial) to expand into a large-scale trial (e.g. a Phase III trial). An intermediate analysis is carried out, in order to decide whether to continue in the same phase or to expand (change) to the next phase, as well as to select those treatments that will continue in the next phase. The target variable may not be the same in the different phases, but at the end of the study both are evaluated.
Multi-Arm Multi-Stage (MAMS)	The MAMS design is used to evaluate multiple experimental treatments against a common control arm in several stages. In general, they add the flexibility to drop non-promising treatments and allow for the addition of new treatments as they become available.
Platform Trial	It is a randomised, adaptive trial, potentially without a scheduled termination date, that makes it possible to assess several interventions in a pathology. It might evolve by adding or discontinuing treatment arms according to pre-established rules. Platform trials involve the establishment of an underlying infrastructure to compare multiple arms, simultaneously or one after the other, to a common control group. The trial is governed by a master protocol that evolves by amendments or additions of successive sub-protocols for each new intervention.

To contextualize these designs and obtain illustrative examples, two separate literature searches were conducted to identify: (1) methodological articles on ADCTs, and (2) applied research articles on ADCTs implemented during the COVID-19 pandemic.

For the first literature search, all research articles published between 2021 and 2022 in the scientific journal “Statistics in Medicine” were screened to identify methodological articles on ADCTs. The journal was chosen for its methodological character when applying statistics to medicine. All selected articles were manually evaluated by two authors focusing on the title and the overview. Any disagreements were solved by consensus. The search was conducted via Medline (PubMed), which is detailed in SUPPLEMENT 1.

The second search regarding COVID-19 ADCTs was conducted via Medline (PubMed) using “Randomized Controlled Trial” words, Mesh term about Clinical Trials and COVID-19 and name of type of adaptive design. The articles were first selected by title and included after reading the abstract, and identified adaptive design.

## 3 Steps for developing the visual framework of ADCTs

A GATE frame [15] representation can be constructed by identifying the key elements of the structured abstracts regarding trial design, methods, results, and conclusions, following item 1b of the CONSORT expanded checklists [1] and ACE statements [5]. The abstract will provide the following information [8], [15], [16]: (1) Identification of the population and eligibility criteria from the Methods section. It should be noted that the population is categorized into the source population (the population from which participants were selected), the eligible population (those who meet the study eligibility criteria), and the participant population (the patients who agreed to take part in the clinical trial, i.e., the recruited population); (2) interventions and comparators intended for each group in the methods, and the number of participants randomised to each group in the results; and (3) primary outcomes in the methods and results sections. The analysis must also account for the existence of interim analyses or adaptive designs that may modify the three aforementioned sections or elements such as randomization or overall clinical trial design. As clinical trial summaries may not include all elements, researchers should examine the clinical trial design (item 9), the eligibility criteria (items 12a and 12b), the intervention and comparator (item 13), the outcomes (item 14), the sample size (item 16b), and the randomization (item 17b) within the randomised clinical trial report, according to the CONSORT checklist [1]. Reviewing the protocol and statistical analysis plan, which can be obtained via the trial registry identifying numbers corresponding to the new Open Science item 2, is recommended.

For each element of the GATE frame, the following must be extracted: the source, eligibility criteria and participant population, the number of participants randomised and the number of participants analysed in each arm, the results for each group, and the estimated effect size along with its precision.

**Fig. 1:**
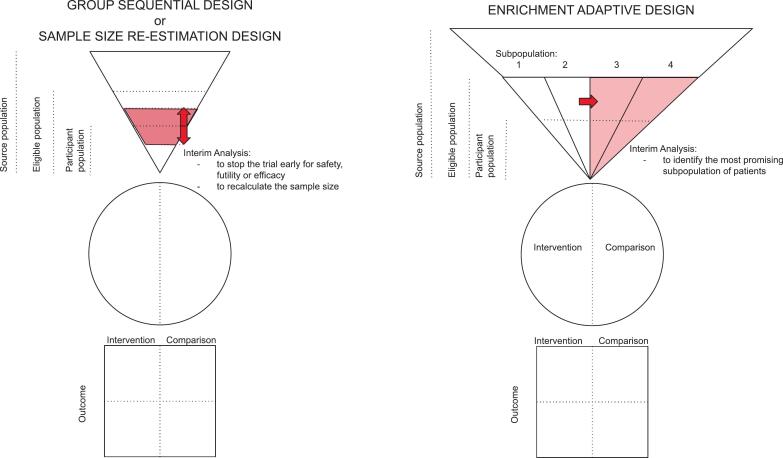
Group sequential methods, sample size re-estimation and enrichment adaptive design.

**Fig. 2:**
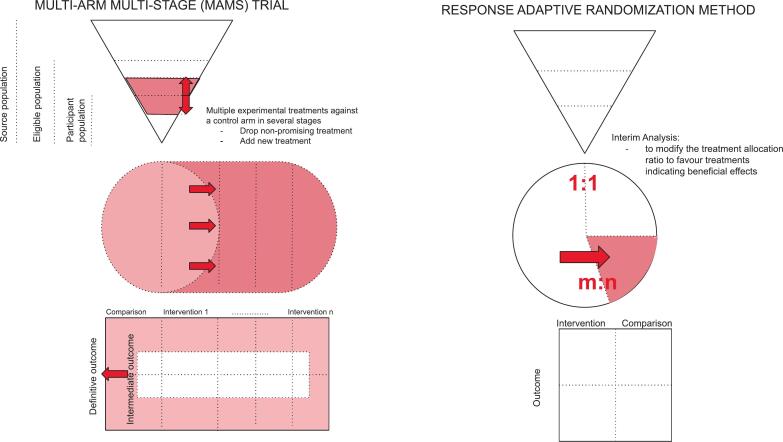
Response adaptive randomization design and Multi-Arms Multi-Stages (MAMS) clinical trials.

Ultimately, both the pre- and post-adaptation GATE frame figures must be presented in cases where a trial adaptation was implemented. In the absence of adaptation, the baseline figure should be retained. Fig. 1 and Fig. 2 illustrate common ADCTs.

## 4 Examples

A total of 812 articles were published in the journal “Statistics in Medicine” during 2021 and 2022. The number of methodological articles on ADCTs was 55 (6,77%). The references to the selected articles can be found in SUPPLEMENT 2, as well as the design type in which they were classified.

### 4.1 Group sequential method example

**Fig. 3:**
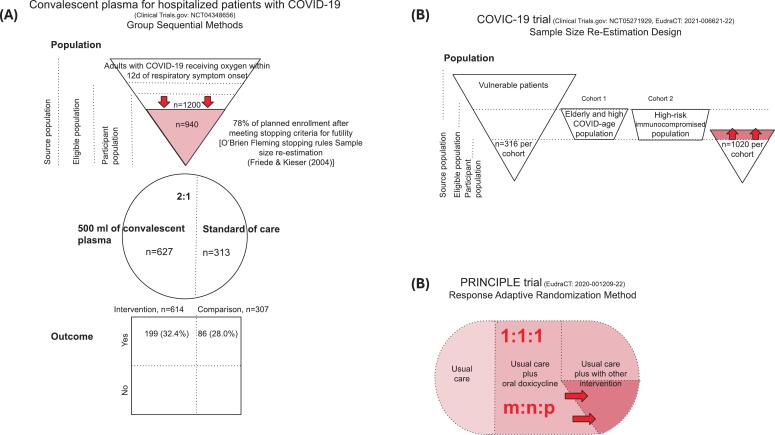
A: the GATE frame for the Group Sequential Method of CONCOR-1 trial. B: the GATE frame for the sample size re-estimation design of COVIC-19 trial. C: the GATE frame for the Response Adaptive Randomization (RAR) method of PRINCIPLE trial.

The CONCOR-1 trial [18] is an open-label, phase 3, randomised controlled trial with a 2:1 allocation ratio. It aims to evaluate the effect of convalescent plasma in adults hospitalized with COVID-19 respiratory illness requiring supplemental oxygen, focusing on 30-day mortality and intubation rates. A single interim analysis was planned once the primary outcome (death or intubation at Day 30) was available for 50% of the target sample, utilizing an O'Brien-Fleming stopping rule. The protocol and statistical analysis plan also included a blinded sample size re-estimation during the interim analysis (Fig. 3A).

### 4.2 Sample size *Re*-estimation design example

The COVIC-19 clinical trial [19] is a multicenter, international, randomised, 1:1, open-label adaptive superiority phase III trial, which aims to evaluate convalescent plasma collected from donors with high-titre neutralising SARS-CoV-2 antibodies plus standard of care (SoC); versus SoC intended to prevent hospitalisation due to progressive COVID-19, or death by day 28 after randomization in vulnerable population, elderly age or high-risk immunocompromised population. The trial approached a sample size re-estimation, justified by the uncertainty existing upon the real risk of serious COVID-19 for the population due to the constant evolution of the SARS-CoV-2 pandemic and the outbreak of new variants. The protocol suggested that the required sample size of 340 per cohort could change and reach up to 1020 per cohort when carrying out an intermediate analysis with 30% of the patients having reached the primary endpoint. Fig. 3B shows the COVID-19 trial modification through the GATE frame. As shown, the bottom part of the triangle, which represents the study's participant population, experienced a three-fold increase. The adaptive nature of the study and the modified parameter are apparent.

### 4.3 Response adaptive randomization method example

The PRINCIPLE trial [20] consists of an adaptive platform randomised trial, national, open-label, multi-arm of interventions against COVID-19 in older people across primary care centres in the United Kingdom. Its goal is to evaluate the usual care plus oral doxicycline, usual care plus with other interventions and usual care plus with regards to the time to first self-reported recovery, and hospitalisation or death related to COVID-19, both measured over 28 days from randomization. The platform trial considered the possibility of including the RAR method if there are at least two active interventions in the trial. The usual care arm will have a fixed allocation of 1/Z. The remaining (Z − 1)/Z allocation probability will be divided among the intervention arms based on interim RAR probabilities. The purpose of RAR is to allocate more participants to the intervention arms with the best observed outcomes (relative to usual care). In Fig. 3C, the PRINCIPLE trial modification is shown: in the circle part of the GATE frame, the line separating the intervention arms was moved to the right as more patients could be allocated to one arm. The RAR design may not be widely known among clinical researchers or clinicians interested in COVID-19 research, but by using this widely known frame, the adaptive nature of the RAR design is easier to understand.

### 4.4 Seamless design (2-in-1 design) example

**Fig. 4:**
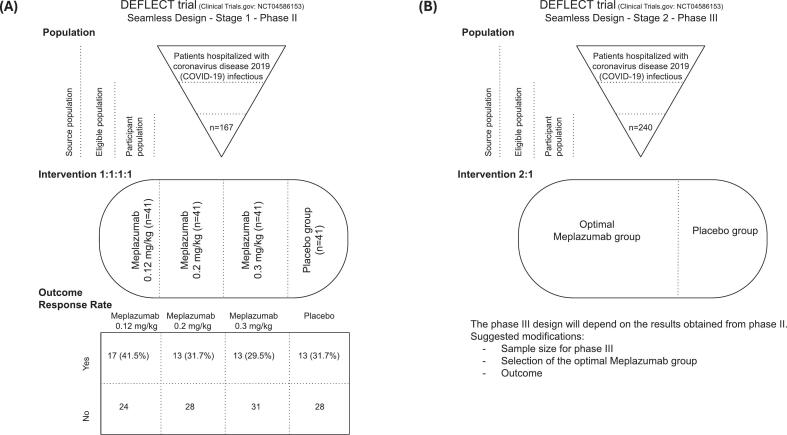
Example DEFLECT trial.

The DEFLECT trial [21] targets a population of hospitalized adults with severe COVID-19 developed under a seamless phase II/III design. In this case, there are two clinical trials: the first is a phase II trial and the second is a phase III trial. Phase III is developed according to what has been found in phase II. Modifications will be related to the estimation of the sample size, to the definition of the target variable and to the selected treatment; that is to say, reducing from 4 treatments to 2. For the graphical explanation (Fig. 4) of phase II, a target variable was selected and used to define the sample size: response rate at day 29 as defined by a sustained improvement of 2 points on a 6-point ordinal scale. The treatments used in phase II are: meplazumab high dose + Standard of Care (SoC), meplazumab medium dose + SoC, meplazumab low dose + SoC, and meplazumab placebo + SoC.

### 4.5 Platform trial example

**Fig. 5:**
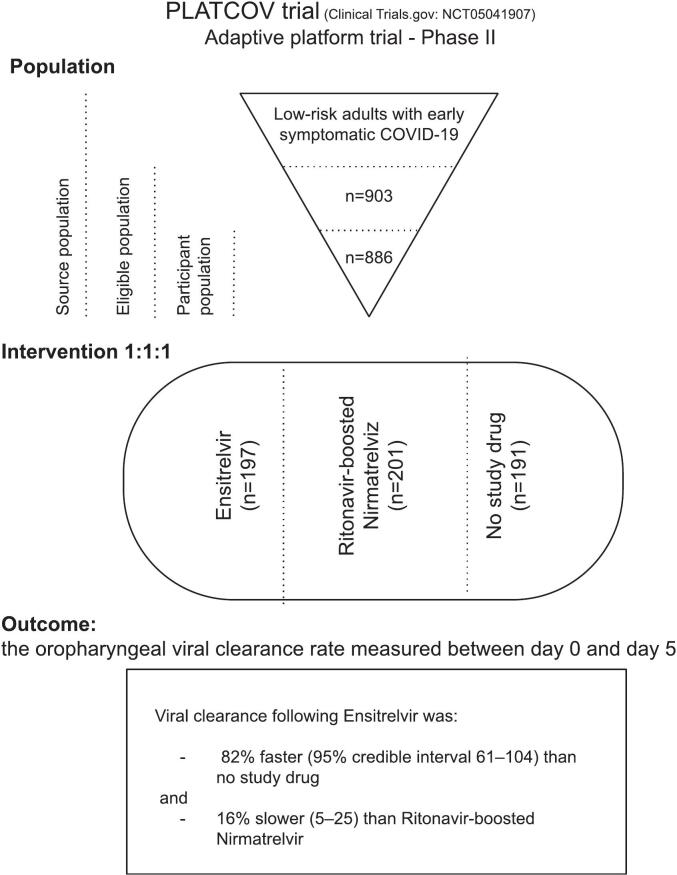
Platform trial: example PLATCOV trial.

The PLATCOV trial [22] is an open-label, phase 2, randomised, controlled, adaptive pharmacometric platform trial [12], [14], [23] enrolling low-risk adult outpatients aged 18–60 years presenting with early symptomatic COVID-19 within 4 days of symptom onset. The primary endpoint was the oropharyngeal SARS-CoV-2 viral clearance rate assessed from day 0 through day 5 comparing eight treatment groups, including oral Ensitrelvir and oral Ritonavir-boosted Nirmatrelvir at standard doses (Fig. 5).

### 4.6 Others complex trial designs examples

The GATE frame can be used in other complex trial designs [23], [24], for instance, basket trials [23], [24], which evaluate the same treatment in several diseases or disease subtypes, and umbrella trials [23], [24], which evaluate different treatments in the same disease characterised by different subtypes potentially sensitive to those treatments. Sometimes, these clinical trials can also incorporate modifications to design aspects during the ongoing study based on pre-planned opportunities to utilize accumulating trial data (ADCTs). The examples presented below do not utilize an adaptive design.

#### 4.6.1 Basket trial example

**Fig. 6:**
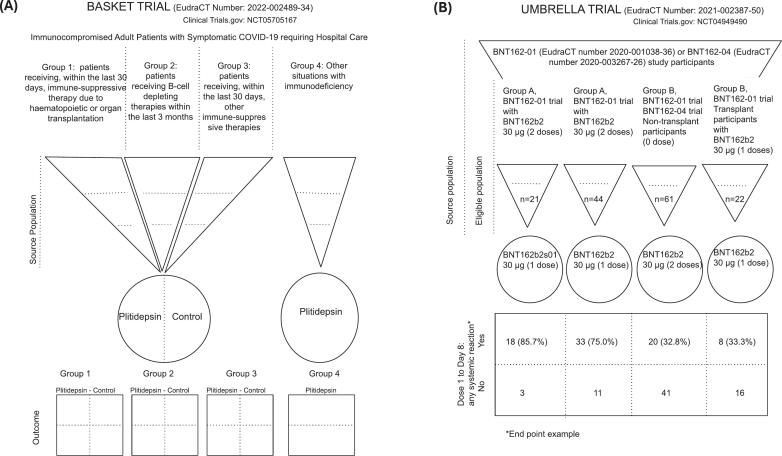
Basket and umbrella trials.

The NEREIDA trial [25] is a multicentre, open-label, randomised, controlled, phase II basket trial designed to evaluate the efficacy and safety of plitidepsin versus control in four distinct cohorts from different source populations of immunocompromised adult patients with symptomatic COVID-19 requiring hospitalisation. This basket trial has a non-adaptive design and is shown in Fig. 6A.

#### 4.6.2 Umbrella trial example

The clinical trial with EudraCT number 2021–002387-50 (NCT04949490) is an umbrella [26], phase II, open-label, rollover trial designed to evaluate the safety and immunogenicity of the following regimens: one booster dose of BNT162b2 (Comirnaty) in BNT162–01 trial subjects, excluding transplant patients, who previously received two 30 μg injections of BNT162b2 (Comirnaty); one booster dose of BNT162b2s01 in BNT162–01 trial subjects, excluding transplant patients, who previously received two 30 μg injections of BNT162b2 (Comirnaty); two booster doses of BNT162b2 (Comirnaty) in BNT162–01 trial subjects, excluding transplant patients, or BNT162–04 trial subjects who did not receive the full two-dose regimen of 30 μg BNT162b2 (Comirnaty); and one booster dose of BNT162b2 (Comirnaty) in BNT162–01 trial transplant patients who previously received only a single injection of 30 μg BNT162b2 (Comirnaty). This umbrella trial has no adaptive design. As shown in Fig. 6B, the umbrella trial scheme depicts the incorporation of different patient subtypes into the eligible population alongside the evaluation of different treatments in the intervention and comparison arms.

## 5 Discussion

The GATE frame [15] and the PICO terminology [16] can complement the ACE guidelines [1], [5] by serving as a teaching tool to explain adaptive design clinical trials to a clinical or nonspecialist audience and promote the interpretation and understanding.

### 5.1 Strengths and weaknesses

We have used the widely used PICO frame [16] and focused on nuclear aspects [8] of clinical trials: population, randomised allocation, arms, outcomes, and phase of the clinical trial. Our approach to understanding ADCTs is compatible with other approaches [13], and graphic representation as the sample size re-estimation in the PHOENIX trial [4], the main target modification in the EXAMINE trial [4], or the use of the Response Adaptive Randomization Method in the TROXACITABINE trial [7]. This taxonomy based on the PICO frame, i.e. focusing on what they are modifying, allows for the integration of those designs contained in more complex designs, for instance, group sequential methods included in MAMS designs, which by definition modify a higher number of elements [27]. And in this simplification, the detail of the mathematical method should be avoided when looking for the design target. A difference was not made in the study either between the bayesian and the frequentist methodology; it was only evaluated what main part of the clinical trial was modified. Thanks to this simplification, we can understand that the same name is used for different scenarios. By way of illustration, “seamless design” is used in clinical trial designs made of two clinical trials, where the performance and key parts of the second trial depend on the first one [21], [28], but where patient data was not analysed together [21] as it can be done in the seamless design or the 2-in-1 design [29], [30]. On the other hand, platform trials are inherently adaptive and offer flexible features such as dropping treatments for futility, graduating treatments to confirmatory trials, or adding new treatments to be tested during the course of a trial [12], [14], [23], [24]. However, it is interesting to identify what these trials are modifying in the clinical trial design when it comes to describing them [31]. Each ADCTs can be further graphically elaborated and developed with its various nuances, but we should be careful because oversimplification could obscure the critical difference in the purpose of ADCTs, the statistical machinery, and the regulatory implications.

One of the limitations of this study is that the bibliography check was carried out in a single scientific journal covering a two-years term. Nevertheless, the selected journal is a methodological journal that values the applicability of statistics in clinical practice, which is useful for the transparent report character of the figures presented within the Evidence-Based Medicine framework [15]. Furthermore, the identified examples were linked to ADCTs to illustrate the application of the GATE frame, including their respective references: NCT (Number of ClinicalTrials.gov) or EUDRACT (European Union Drug Regulating Authorities Clinical Trials), within the SANRA (Scale for the Quality Assessment of Narrative Review Articles) domain [32]. Furthermore, it should be noted that we do not present any pedagogical evaluation of the GATE frame [15]. On the other hand, the development of the figure is a process of verifying the associated items of the CONSORT guideline [1] and ACE checklist [5], and therefore, it could enhance the reporting quality.

### 5.2 Use and future research

The GATE frame [15] and the PICO terminology [16] are useful tools in ADCTs because they promote transparency and clarity in scientific papers concerning clinical trials, by indicating the minimum number of elements to be presented and the implication of the adaptive design for these elements. Furthermore, using the GATE frame to present ADCTs eases the critical evaluation, the research waste caused by poor reports, the interpretation of results and the transfer of clinical findings from the research. Scientific transparency enables public and patient access to, and participation in [10], the research process, thereby contributing to improvements in overall population health and to response to emerging infectious diseases. In addition, understanding ADCTs helps clinical researchers identify the best design, whether adaptive or not, to answer the research question, as well as it fosters the interaction between methodologic and clinical researchers. These are recommended tools complementing the CONSORT [1] and ACE [5] guidelines.

## Authors' contribution

Conceptualization: DL, ALB, GMC, AGR, MR-R, EJC, MM-O.

Formal analysis: DL, MM-O.

Funding acquisition: DL, ALB, GMC, AGR, MR-R, EJC, MM-O.

Investigation: DL, ALB, GMC, AGR, MR-R, EJC, MM-O.

Methodology: DL, EJC, MM-O.

Supervision: EJC, MM-O.

Visualization: DL, MM-O.

Writing - original draft: DL.

Writing - review & editing: DL, ALB, GMC, AGR, MR-R, EJC, MM-O.

All authors read and approved the final manuscript.

## CRediT authorship contribution statement

**David Lora:** Writing – review & editing, Writing – original draft, Visualization, Methodology, Investigation, Funding acquisition, Formal analysis, Conceptualization. **Antonio Lalueza Blanco:** Writing – review & editing, Investigation, Funding acquisition, Conceptualization. **Guillermo Maestro de la Calle:** Writing – review & editing, Investigation, Funding acquisition, Conceptualization. **Ana García Reyne:** Writing – review & editing, Investigation, Funding acquisition, Conceptualization. **María Ruíz-Ruigómez:** Writing – review & editing, Investigation, Funding acquisition, Conceptualization. **Enrique J. Calderón:** Writing – review & editing, Supervision, Methodology, Investigation, Funding acquisition, Conceptualization. **Miguel Menéndez-Orenga:** Writing – review & editing, Visualization, Supervision, Methodology, Investigation, Funding acquisition, Formal analysis, Conceptualization.

## Consent for publication

Not applicable.

## Ethics approval and consent to participate

Not applicable.

## Funding

This study was funded by the Instituto de Salud Carlos III (ISCIII) through the project “PI21/01815” and co-funded by the European Union. MR-R holds a research contract “Juan Rodés” (JR22/00051) from the Spanish Ministry of Science, Innovation and Universities, Instituto de Salud Carlos III.

## Declaration of competing interest

The authors declare that they have no competing interests.

## Data Availability

Not applicable.

## Appendix A Supplementary data

Search strategy and classification of methodological articles on adaptive design clinical trials
