# Supplementary material for: A visual framework for classifying adaptive design clinical trials using the GATE frame and PICO terminology
Source: Dialogues Health. 2026 May 28;8:100315. doi: 10.1016/j.dialog.2026.100315 (PMC13253079; doi:10.1016/j.dialog.2026.100315)
Supplement: Supplementary file 1 — Search strategy and classification of methodological articles on adaptive design clinical trials [file mmc1.docx]

**SUPPLEMENT**

**SUPPLEMENT 1. Search strategy**

Search strategy for Medline (PubMed):

Date search: March 2026.

Number of references found: 812.

Search code: PubMed.

| Search in String format | | |
| --- | --- | --- |
| #1 | ("Statistics in medicine"[Journal]) AND (2021:2022[pdat]) | 812 |

Supplement Table 1. Search strategy for Medline (PubMed)

**SUPPLEMENT 2. Articles of adaptive design clinical trials**

| Title | Type of adaptive design |
| --- | --- |
| Shrestha S, Jain S. A Bayesian-bandit adaptive design for N-of-1 clinical trials. Stat Med. 2021 Mar 30;40(7):1825-1844. doi: 10.1002/sim.8873. Epub 2021 Jan 18. PMID: 33462851. | Response Adaptive Randomization Method |
| Hartman H, Tamura RN, Schipper MJ, Kidwell KM. Design and analysis considerations for utilizing a mapping function in a small sample, sequential, multiple assignment, randomized trials with continuous outcomes. Stat Med. 2021 Jan 30;40(2):312-326. doi: 10.1002/sim.8776. Epub 2020 Oct 27. PMID: 33111381; PMCID: PMC9109654. | Response Adaptive Randomization Method |
| Fang F, Hochstedler KA, Tamura RN, Braun TM, Kidwell KM. Bayesian methods to compare dose levels with placebo in a small n, sequential, multiple assignment, randomized trial. Stat Med. 2021 Feb 20;40(4):963-977. doi: 10.1002/sim.8813. Epub 2020 Nov 20. PMID: 33216360. | Response Adaptive Randomization Method |
| Lane A. Conditional information and inference in response-adaptive allocation designs. Stat Med. 2022 Jan 30;41(2):390-406. doi: 10.1002/sim.9243. Epub 2021 Nov 8. PMID: 34747523. | Response Adaptive Randomization Method  Dose-Finding Design |
| Frieri R, Zagoraiou M. Optimal and ethical designs for hypothesis testing in multi-arm exponential trials. Stat Med. 2021 May 20;40(11):2578-2603. doi: 10.1002/sim.8919. Epub 2021 Mar 9. PMID: 33687086. | Response Adaptive Randomization Method |
| Matsuura K, Honda J, El Hanafi I, Sozu T, Sakamaki K. Optimal adaptive allocation using deep reinforcement learning in a dose-response study. Stat Med. 2022 Mar 30;41(7):1157-1171. doi: 10.1002/sim.9247. Epub 2021 Nov 7. PMID: 34747043; PMCID: PMC9298337. | Response Adaptive Randomization Method |
| Grayling MJ, Wason JMS, Villar SS. Response adaptive intervention allocation in stepped-wedge cluster randomized trials. Stat Med. 2022 Mar 15;41(6):1081-1099. doi: 10.1002/sim.9317. Epub 2022 Jan 21. PMID: 35064595; PMCID: PMC7612601. | Response Adaptive Randomization Method in Stepped-wedge cluster randomized trial |
| Zhan T, Cui L, Geng Z, Zhang L, Gu Y, Chan ISF. A practical response adaptive block randomization (RABR) design with analytic type I error protection. Stat Med. 2021 Oct 15;40(23):4947-4960. doi: 10.1002/sim.9104. Epub 2021 Jun 10. PMID: 34111902. | Response Adaptive Randomization Method |
| Yu Y, Xu C, Zhong J, Cheung SH. Comparison of treatments with ordinal responses in trials with sequential monitoring and response-adaptive randomization. Stat Med. 2022 Nov 10;41(25):5061-5083. doi: 10.1002/sim.9554. Epub 2022 Aug 16. PMID: 35973712. | Response Adaptive Randomization Method  Group Sequential Method |
| Su PF. Response-adaptive treatment allocation for clinical studies with recurrent event and terminal event data. Stat Med. 2022 Jan 30;41(2):258-275. doi: 10.1002/sim.9235. Epub 2021 Oct 24. PMID: 34693543. | Response Adaptive Randomization Method |
| Li X, Ma W, Hu F. Sample size re-estimation for covariate-adaptive randomized clinical trials. Stat Med. 2021 May 30;40(12):2839-2858. doi: 10.1002/sim.8939. Epub 2021 Mar 17. PMID: 33733513. | Sample size re-estimation method  Covariate Adaptive Randomization Method |
| Mehta C, Bhingare A, Liu L, Senchaudhuri P. Optimal adaptive promising zone designs. Stat Med. 2022 May 20;41(11):1950-1970. doi: 10.1002/sim.9339. Epub 2022 Feb 14. PMID: 35165917. | Sample Size Re-Estimation Design |
| Igeta M, Matsui S. Blinded sample size re-estimation for comparing over-dispersed count data incorporating follow-up lengths. Stat Med. 2022 Dec 20;41(29):5622-5644. doi: 10.1002/sim.9584. Epub 2022 Sep 23. PMID: 36148560. | Sample Size Re-Estimation Design |
| Zhou J, Jiang X, Xia HA, Wei P, Hobbs BP. Predicting outcomes of phase III oncology trials with Bayesian mediation modeling of tumor response. Stat Med. 2022 Feb 20;41(4):751-768. doi: 10.1002/sim.9268. Epub 2021 Dec 9. PMID: 34888892. | Sample Size Re-Estimation Design  Group Sequential Method |
| Lawrence J. Sample size adaptations and evaluation of pediatric study interpretability. Stat Med. 2021 Sep 30;40(22):4809-4814. doi: 10.1002/sim.9096. Epub 2021 Jun 8. PMID: 34102704. | Sample Size Re-Estimation Design |
| Wang P, Chow SC. Sample size re-estimation in clinical trials. Stat Med. 2021 Nov 30;40(27):6133-6149. doi: 10.1002/sim.9175. Epub 2021 Aug 25. PMID: 34433225. | Sample Size Re-Estimation Design |
| Kunzmann K, Grayling MJ, Lee KM, Robertson DS, Rufibach K, Wason JMS. Conditional power and friends: The why and how of (un)planned, unblinded sample size recalculations in confirmatory trials. Stat Med. 2022 Feb 28;41(5):877-890. doi: 10.1002/sim.9288. Epub 2022 Jan 13. PMID: 35023184; PMCID: PMC9303654. | Sample Size Re-Estimation Design |
| Li W, Bai X, Deng Q, Liu F, Chen C. Estimation of treatment effect in 2-in-1 adaptive design and some of its extensions. Stat Med. 2021 May 20;40(11):2556-2577. doi: 10.1002/sim.8917. Epub 2021 Mar 15. Erratum in: Stat Med. 2023 Oct 15;42(23):4299. PMID: 33723865. | Seamless Design (2-in-1 Design) |
| Takahashi K, Ishii R, Maruo K, Gosho M. Statistical tests for two-stage adaptive seamless design using short- and long-term binary outcomes. Stat Med. 2022 Sep 20;41(21):4130-4142. doi: 10.1002/sim.9500. Epub 2022 Jun 17. PMID: 35713225. | Seamless Design (2-in-1 Design) |
| Khan JN, Kimani PK, Glimm E, Stallard N. Adjusting for treatment selection in phase II/III clinical trials with time to event data. Stat Med. 2023 Jan 30;42(2):146-163. doi: 10.1002/sim.9606. Epub 2022 Nov 23. PMID: 36419206; PMCID: PMC10098876. | Seamless Design (2-in-1 Design) |
| Serra A, Mozgunov P, Jaki T. An order restricted multi-arm multi-stage clinical trial design. Stat Med. 2022 Apr 30;41(9):1613-1626. doi: 10.1002/sim.9314. Epub 2022 Jan 19. PMID: 35048391; PMCID: PMC7612618. | MAMS |
| Mu R, Xu J, Tang RS, Kopetz S, Yuan Y. A Bayesian phase I/II platform design for co-developing drug combination therapies for multiple indications. Stat Med. 2022 Jan 30;41(2):374-389. doi: 10.1002/sim.9242. Epub 2021 Nov 3. PMID: 34730248. | Platform design  Dose-Finding Design |
| Mu R, Pan H, Xu G. A Bayesian adaptive phase I/II platform trial design for pediatric immunotherapy trials. Stat Med. 2021 Jan 30;40(2):382-402. doi: 10.1002/sim.8780. Epub 2020 Oct 22. PMID: 33094528. | Adaptive Platform design: Dose-Finding Design, Response Adaptive Randomization Method, seamless design, Group Sequential Method |
| Liu Y, Kane M, Esserman D, Blaha O, Zelterman D, Wei W. Bayesian local exchangeability design for phase II basket trials. Stat Med. 2022 Sep 30;41(22):4367-4384. doi: 10.1002/sim.9514. Epub 2022 Jul 1. PMID: 35777367; PMCID: PMC10279458. | Sequential Design in basket trial |
| Takeda K, Liu S, Rong A. Constrained hierarchical Bayesian model for latent subgroups in basket trials with two classifiers. Stat Med. 2022 Jan 30;41(2):298-309. doi: 10.1002/sim.9237. Epub 2021 Oct 25. PMID: 34697822. | Sequential Design in basket trial |
| Guo B, Zang Y. BIPSE: A biomarker-based phase I/II design for immunotherapy trials with progression-free survival endpoint. Stat Med. 2022 Mar 30;41(7):1205-1224. doi: 10.1002/sim.9265. Epub 2021 Nov 25. PMID: 34821409; PMCID: PMC9335906. | Dose-Finding Design  Response Adaptive Randomization Method |
| Mozgunov P, Jaki T, Gounaris I, Goddemeier T, Victor A, Grinberg M. Practical implementation of the partial ordering continual reassessment method in a Phase I combination-schedule dose-finding trial. Stat Med. 2022 Dec 30;41(30):5789-5809. doi: 10.1002/sim.9594. Epub 2022 Nov 25. PMID: 36428217; PMCID: PMC10100035. | Dose-Finding Design |
| Zhou Y, Lin R, Lee JJ, Li D, Wang L, Li R, Yuan Y. TITE-BOIN12: A Bayesian phase I/II trial design to find the optimal biological dose with late-onset toxicity and efficacy. Stat Med. 2022 May 20;41(11):1918-1931. doi: 10.1002/sim.9337. Epub 2022 Jan 31. PMID: 35098585; PMCID: PMC9199061. | Dose-Finding Design |
| Barnett H, Boix O, Kontos D, Jaki T. Dose finding studies for therapies with late-onset toxicities: A comparison study of designs. Stat Med. 2022 Dec 30;41(30):5767-5788. doi: 10.1002/sim.9593. Epub 2022 Oct 17. PMID: 36250912; PMCID: PMC10092569. | Dose-Finding Design |
| Braun TM, Mercier F. Extending the Continual Reassessment Method to accommodate step-up dosing in Phase I trials. Stat Med. 2022 Sep 10;41(20):3975-3990. doi: 10.1002/sim.9487. Epub 2022 Jun 5. PMID: 35662077; PMCID: PMC9546169. | Dose-Finding Design |
| Kojima M. Early completion of phase I cancer clinical trials with Bayesian optimal interval design. Stat Med. 2021 Jun 30;40(14):3215-3226. doi: 10.1002/sim.8886. Epub 2021 Apr 12. PMID: 33844323 | Dose-Finding Design |
| Shi H, Cao J, Yuan Y, Lin R. uTPI: A utility-based toxicity probability interval design for phase I/II dose-finding trials. Stat Med. 2021 May 20;40(11):2626-2649. doi: 10.1002/sim.8922. Epub 2021 Mar 2. PMID: 33650708. | Dose-Finding Design |
| Curtis A, Smith B, Chapple AG. Subgroup-specific dose finding for phase I-II trials using Bayesian clustering. Stat Med. 2022 Jul 20;41(16):3164-3179. doi: 10.1002/sim.9410. Epub 2022 Apr 16. PMID: 35429178; PMCID: PMC9324955. | Dose-Finding Design |
| Lee J, Thall PF, Msaouel P. Precision Bayesian phase I-II dose-finding based on utilities tailored to prognostic subgroups. Stat Med. 2021 Oct 30;40(24):5199-5217. doi: 10.1002/sim.9120. Epub 2021 Jul 9. PMID: 34636054; PMCID: PMC9175509. | Dose-Finding Design |
| Biard L, Lee SM, Cheng B. Seamless phase I/II design for novel anticancer agents with competing disease progression. Stat Med. 2021 Sep 20;40(21):4568-4581. doi: 10.1002/sim.9080. Epub 2021 Jul 2. PMID: 34213022; PMCID: PMC9202313. | Seamless Design (2-in-1 Design)  Dose-Finding Design |
| Lake SL, Quintana MA, Broglio K, Panagoulias J, Berry SM, Panzara MA. Bayesian adaptive design for clinical trials in Duchenne muscular dystrophy. Stat Med. 2021 Aug 30;40(19):4167-4184. doi: 10.1002/sim.9021. Epub 2021 May 7. Erratum in: Stat Med. 2022 Apr 30;41(9):1733-1734. PMID: 33960507. | Group Sequential Method |
| Tsiatis AA, Davidian M. Group sequential methods for interim monitoring of randomized clinical trials with time-lagged outcome. Stat Med. 2022 Dec 10;41(28):5517-5536. doi: 10.1002/sim.9580. Epub 2022 Sep 18. PMID: 36117235; PMCID: PMC9825950. | Group Sequential Method |
| Chen Y, Lawrence J, Lee MT. Group sequential design for randomized trials using "first hitting time" model. Stat Med. 2022 Jun 15;41(13):2375-2402. doi: 10.1002/sim.9360. Epub 2022 Mar 10. PMID: 35274361. | Group Sequential Method |
| Li B, Su L, Ye Y, Yan F. M&M: A maximum duration design with the Maxcombo test for a group sequential trial of an immunotherapy with a random delayed treatment effect. Stat Med. 2022 Feb 20;41(4):815-830. doi: 10.1002/sim.9251. Epub 2021 Nov 15. PMID: 34783047. | Group Sequential Method |
| Tamhane AC, Xi D, Gou J. Group sequential Holm and Hochberg procedures. Stat Med. 2021 Oct 30;40(24):5333-5350. doi: 10.1002/sim.9128. Epub 2021 Jul 8. PMID: 34636081. | Group Sequential Method |
| Wang J, Ma J, Cai C, Daver N, Ning J. A Bayesian hierarchical monitoring design for phase II cancer clinical trials: Incorporating information on response duration into monitoring rules. Stat Med. 2021 Sep 20;40(21):4629-4639. doi: 10.1002/sim.9084. Epub 2021 Jun 7. PMID: 34101217; PMCID: PMC8376777. | Group Sequential Method |
| R Silva I, Maro J, Kulldorff M. Exact sequential test for clinical trials and post-market drug and vaccine safety surveillance with Poisson and binary data. Stat Med. 2021 Sep 30;40(22):4890-4913. doi: 10.1002/sim.9094. Epub 2021 Jun 13. PMID: 34120357; PMCID: PMC8441767. | Group Sequential Method |
| Pilz M, Kunzmann K, Herrmann C, Rauch G, Kieser M. Optimal planning of adaptive two-stage designs. Stat Med. 2021 Jun 15;40(13):3196-3213. doi: 10.1002/sim.8953. Epub 2021 Mar 18. PMID: 33738842. | Group Sequential Method |
| Kotalik A, Vock DM, Hobbs BP, Koopmeiners JS. A group-sequential randomized trial design utilizing supplemental trial data. Stat Med. 2022 Feb 20;41(4):698-718. doi: 10.1002/sim.9249. Epub 2021 Nov 9. PMID: 34755388; PMCID: PMC8795487. | Group Sequential Method |
| Ren T, Shen W, Zhang L, Zhao H. Bayesian phase II clinical trial design with noncompliance. Stat Med. 2021 Sep 10;40(20):4457-4472. doi: 10.1002/sim.9041. Epub 2021 May 28. PMID: 34050539. | Group Sequential Method |
| Lloyd CJ. Exact confidence limits after a group sequential single arm binary trial. Stat Med. 2021 May 10;40(10):2389-2399. doi: 10.1002/sim.8909. Epub 2021 Mar 2. PMID: 33650191. | Group Sequential Method |
| Kundu MG, Sarkar J. On information fraction for Fleming-Harrington type weighted log-rank tests in a group-sequential clinical trial design. Stat Med. 2021 May 10;40(10):2321-2338. doi: 10.1002/sim.8905. Epub 2021 Feb 24. PMID: 33624861. | Group Sequential Method |
| Meis J, Pilz M, Herrmann C, Bokelmann B, Rauch G, Kieser M. Optimization of the two-stage group sequential three-arm gold-standard design for non-inferiority trials. Stat Med. 2023 Feb 20;42(4):536-558. doi: 10.1002/sim.9630. Epub 2022 Dec 28. PMID: 36577519. | Group Sequential Method |
| Schoenfeld DA, Ramchandani R, Finkelstein DM. Designing a longitudinal clinical trial based on a composite endpoint: Sample size, monitoring, and adaptation. Stat Med. 2022 Oct 30;41(24):4745-4755. doi: 10.1002/sim.9416. Epub 2022 Jul 11. PMID: 35818331. | Group Sequential Method  Sample size re-estimation |
| Robertson DS, Choodari-Oskooei B, Dimairo M, Flight L, Pallmann P, Jaki T. Point estimation for adaptive trial designs I: A methodological review. Stat Med. 2023 Jan 30;42(2):122-145. doi: 10.1002/sim.9605. Epub 2022 Nov 30. PMID: 36451173; PMCID: PMC7613995. | Methodology |
| Di Stefano F, Pannaux M, Correges A, Galtier S, Robert V, Saint-Hilary G. A comparison of estimation methods adjusting for selection bias in adaptive enrichment designs with time-to-event endpoints. Stat Med. 2022 May 10;41(10):1767-1779. doi: 10.1002/sim.9327. Epub 2022 Jan 31. PMID: 35098579. | Adaptive Enrichment Design |
| Burnett T, Jennison C. Adaptive enrichment trials: What are the benefits? Stat Med. 2021 Feb 10;40(3):690-711. doi: 10.1002/sim.8797. Epub 2020 Nov 26. PMID: 33244786; PMCID: PMC7839594. | Adaptive Enrichment Design |
| Ballarini NM, Burnett T, Jaki T, Jennison C, König F, Posch M. Optimizing subgroup selection in two-stage adaptive enrichment and umbrella designs. Stat Med. 2021 May 30;40(12):2939-2956. doi: 10.1002/sim.8949. Epub 2021 Mar 29. PMID: 33783020; PMCID: PMC8251960. | Adaptive Enrichment Design and Umbrella Designs |
| Johns H, Italiano D, Campbell B, Churilov L. Common scale minimal sufficient balance: An improved method for covariate-adaptive randomization based on the Wilcoxon-Mann-Whitney odds ratio statistic. Stat Med. 2022 May 10;41(10):1846-1861. doi: 10.1002/sim.9332. Epub 2022 Feb 17. PMID: 35176811; PMCID: PMC9303921. | Covariate Adaptive Randomization Method |
| Ma W, Tu F, Liu H. Regression analysis for covariate-adaptive randomization: A robust and efficient inference perspective. Stat Med. 2022 Dec 20;41(29):5645-5661. doi: 10.1002/sim.9585. Epub 2022 Sep 22. PMID: 36134688. | Covariate Adaptive Randomization Method |
